# Bioinformatic Approaches Reveal Metagenomic Characterization of Soil Microbial Community

**DOI:** 10.1371/journal.pone.0093445

**Published:** 2014-04-01

**Authors:** Zhuofei Xu, Martin Asser Hansen, Lars H. Hansen, Samuel Jacquiod, Søren J. Sørensen

**Affiliations:** Section of Microbiology, Department of Biology, University of Copenhagen, Copenhagen, Denmark; Institute for Plant Protection (IPP), CNR, Italy

## Abstract

As is well known, soil is a complex ecosystem harboring the most prokaryotic biodiversity on the Earth. In recent years, the advent of high-throughput sequencing techniques has greatly facilitated the progress of soil ecological studies. However, how to effectively understand the underlying biological features of large-scale sequencing data is a new challenge. In the present study, we used 33 publicly available metagenomes from diverse soil sites (i.e. grassland, forest soil, desert, Arctic soil, and mangrove sediment) and integrated some state-of-the-art computational tools to explore the phylogenetic and functional characterizations of the microbial communities in soil. Microbial composition and metabolic potential in soils were comprehensively illustrated at the metagenomic level. A spectrum of metagenomic biomarkers containing 46 taxa and 33 metabolic modules were detected to be significantly differential that could be used as indicators to distinguish at least one of five soil communities. The co-occurrence associations between complex microbial compositions and functions were inferred by network-based approaches. Our results together with the established bioinformatic pipelines should provide a foundation for future research into the relation between soil biodiversity and ecosystem function.

## Introduction

Soil is considered to be the most diverse natural environment on the Earth [1], [2]. The soil microbial communities harbor thousands of different prokaryotic organisms that contain a substantial number of genetic information, ranging from 2,000 to 18,000 different genomes estimated in one gram of soil [3]. One of the most important issues in the field of soil ecology is to uncover the complex relationships between microbial compositions and functional diversity in soil.

Based on traditional approaches for cultivating and isolating soil microorganisms, early studies have focused on culturable bacteria which only account for less than 1% of soil microbial populations [4]. These studies have already discovered many novel genes encoding interesting enzymes and antimicrobials in soils via functional screens and clone-based Sanger sequencing [1], [5], [6]. Due to the recent advent of High-Throughput Sequencing (HTS) technologies, metagenomic sequencing approaches have been applied to investigate characterizations of diverse soil microbial communities, including target sequencing of the phylogenetic marker gene encoding 16S rRNA [7], [8] and whole-metagenome shotgun sequencing [9]–[13]. However, the majority of 16S rRNA gene-based studies are committed to the interpretation of community composition but poorly focus on the functional and metabolic properties in a microbial community [14]. In addition, integrated bioinformatic analyses for microbial community-level taxonomic affiliation, metabolic reconstruction, and interaction network, seems to be less studied for the highly diverse soil ecosystems. Currently, MG-RAST [15], IMG/M [16], and CAMERA [17] are the major databases that can support deposition and analysis of metagenomic datasets. Uploading large sequencing data and the subsequent analysis jobs on these web servers sometimes take long waiting time and even weeks. The computational pipelines implemented by these prominent platforms are capable of processing many analysis tasks, but some approaches for special biological inference and graphical visualization still need to be complemented [18].

Recently, together with the rapid development of the Human Microbiome Project, numerous computational tools and methodologies have been developed for effective interpretation and visualization of taxonomic and metabolic profiling of complex microbial communities [19], [20] and could be applied to the analysis of the soil microbiota. Particularly, some outstanding computational techniques that could better explain the complexity and heterogeneity of microbial communities are still less applied in the study of the soil microbiota, e.g. prediction of metagenomic biomarkers and network-based correlation analyses [21], [22]. In this study, we aim to explore the characterizations of the soil microbiota through integrating the current state-of-the-art bioinformatics tools. A collection dataset of 33 publicly available soil metagenomes was investigated in a custom metagenomic data mining pipeline for explaining and visualizing microbial compositions and metabolic potential. A full spectrum of metagenomic biomarkers and a network of taxon co-occurrence patterns were inferred to hopefully provide some new insights into the underlying mechanisms of complex ecological relationships in the soil microbial community.

## Materials and Methods

### Ethics statement

No specific permissions were required for the described field studies. The study locations are not privately owned and the field studies did not involve endangered or protected species.

### Collection and quality control of metagenomic datasets

Thirty-three metagenomes sampled from five natural soil environments were publicly available and collected in the present study: 14 from grassland, seven from forest soil, nine from desert, two from Arctic soil, and one from mangrove sediment. The metagenomic datasets used can be downloadable according to the list of sequence accession numbers or web links shown in Table S1. All datasets have been produced by whole-metagenome shotgun sequencing using the Roche 454 or Illumina platforms. More reference information about these chosen metagenomes was listed in Table 1. For the datasets of FASTQ formatted sequence reads without quality control, we performed a quality check of bases by using the package Biopieces (http://www.biopieces.org). Low quality ends per read were trimmed by *trim_seq*. Trimming progressed until all bases in a 3-bp stretch with minimum quality score of 20. High quality reads were retained if satisfying the following criteria: minimum average quality score of 15 in a sliding window of 20 bp; minimum read length of 50 bp.

**Table 1:** Summary of 33 soil metagenomes used in this study.

Biome type	ID	Sequencer	Original name	Location	Coordinates	No. of sequences	No. of bases (Mb)	Reference
Forest soil	F1	454	NA	Puerto Rican	18°18′N, 65°50′W	782,404	322	[9]
Forest soil	F2	454	NA	Massachusetts, USA	42°54′N, 72°18′W	1,439,445	742	[11]
Forest soil	F3	Illumina	AR3	Misiones, Argentina	26°44′S, 54°41′W	5,235,352	524	[12]
Forest soil	F4	Illumina	PE6	Manu National Park, Peru	12°38′S, 71°14′W	9,206,662	921	[12]
Forest soil	F5	Illumina	BZ1	Bonanza Creek, Alaska, USA	64°48′N, 148°15′W	6,543,903	654	[12]
Forest soil	F6	Illumina	CL1	South Carolina, USA	34°37′N, 81°40′W	6,402,940	640	[12]
Forest soil	F7	Illumina	DF1	North Carolina, USA	35°58′N, 79°5′W	3,890,044	389	[12]
Arctic soil	A1	454	Control	Alert, Canada	82°31′N, 62°17′W	495,998	254	[13]
Arctic soil	A2	Illumina	TL1	Toolik Lake LTER, Alaska, USA	68°38′N, 149°35′W	6,011,971	601	[12]
Grassland	G1	454	F1	Hertfordshire, UK	51°48′N, 0°14′E	976,268	358	[10]
Grassland	G2	454	F2a	Hertfordshire, UK	51°48′N, 0°14′E	1,094,883	471	[10]
Grassland	G3	454	F2b	Hertfordshire, UK	51°48′N, 0°14′E	890,966	321	[10]
Grassland	G4	454	F3	Hertfordshire, UK	51°48′N, 0°14′E	754,829	311	[10]
Grassland	G5	454	F4	Hertfordshire, UK	51°48′N, 0°14′E	946,839	391	[10]
Grassland	G6	454	F5	Hertfordshire, UK	51°48′N, 0°14′E	754,135	256	[10]
Grassland	G7	454	F6	Hertfordshire, UK	51°48′N, 0°14′E	782,342	306	[10]
Grassland	G8	454	J1	Hertfordshire, UK	51°48′N, 0°14′E	1,130,719	466	[10]
Grassland	G9	454	J1a	Hertfordshire, UK	51°48′N, 0°14′E	1,137,813	433	[10]
Grassland	G10	454	J1b	Hertfordshire, UK	51°48′N, 0°14′E	919,406	343	[10]
Grassland	G11	454	J1rhizo	Hertfordshire, UK	51°48′N, 0°14′E	1,025,699	369	[10]
Grassland	G12	454	J4	Hertfordshire, UK	51°48′N, 0°14′E	1,135,084	506	[10]
Grassland	G13	454	J7	Hertfordshire, UK	51°48′N, 0°14′E	938,860	339	[10]
Grassland	G14	Illumina	KP1	Kansas, USA	39°6′N, 96°36′W	5,348,832	535	[12]
Desert	D1	Illumina	EB017	Garwood Valley, Antarctica	78°2′S, 163°52′E	7,947,086	795	[12]
Desert	D2	Illumina	EB019	Lake Bonney Valley, Antarctica	77°44′S, 162°18′E	5,454,640	545	[12]
Desert	D3	Illumina	EB020	Lake Fryxell Valley, Antarctica	77°36′S, 163°15′E	9,446,684	945	[12]
Desert	D4	Illumina	EB021	Lake Hoare Valley, Antarctica	77°38′S, 162°53′E	6,543,681	654	[12]
Desert	D5	Illumina	EB024	Wright Valley, Antarctica	77°32′S, 161°42′E	10,863,646	1,086	[12]
Desert	D6	Illumina	EB026	Lake Bonney Valley, Antarctica	77°44′S, 162°19′E	5,951,684	595	[12]
Desert	D7	Illumina	MD3	Mojave Desert, California, USA	34°54′N, 115°39′W	5,899,497	590	[12]
Desert	D8	Illumina	SF2	Chihuahuan Desert, New Mexico, USA	35°23′N, 105°56′W	6,805,456	681	[12]
Desert	D9	Illumina	SV1	Chihuahuan Desert, New Mexico, USA	34°20′N, 106°44′W	11,122,546	1,112	[12]
Mangrove sediment	M1	454	NA	Bertioga, Brazil	23°53′S, 46°12′W	913,752	216	[23]

### Estimation of microbial composition

MetaPhlAn v1.7 [24] and BLAST v2.2.22 [25] were employed for profiling the taxonomic clades in the metagenomic datasets. Briefly, metagenomic reads were firstly mapped to the MetaPhlAn reference database composed of unique clade-specific marker genes using BLASTN. The non-default parameters used for BLASTN sequencing similarity searching were as follows: E-value cutoff of 1e-10, word size of 12, and minimum alignment length of 75 nt. Relative abundance scores at all taxonomic levels from the domain level to the species level were then estimated by MetaPhlAn. In the text, mean values of abundances were shown for the mentioned taxon. To assess the compositional similarity among soil samples from different microbial communities, the Bray-Curtis measure of beta diversity [26] was employed to compare all pairwise taxonomic abundances between each sample-pair using a R function *vegdist* in the package vegan [27]. The permutation-based multivariate analysis of variance (PERMANOVA) and 2D stress value were then estimated. Based on the resultant Bray-Curtis similarity distance matrix, non-metric multidimensional scaling (NMDS) was adopted to visualize the dispersion of community structure. Multivariate analysis was carried out using vegan [27] and R (http://www.R-project.org) [28].

### Metabolic reconstruction of metagenomes

Metabolic reconstruction was carried out using the HUMAnN methodology designed for the functional analyses of meta'omics [29]. High quality reads were initially mapped to the characterized protein functional database KEGG Orthology v54 [30] using the accelerated translated BLAST program USEARCH v6.0.307 [31]. The cutoff E-value was set to 1e-6 and best hits were then used to estimate relative abundances of KEGG orthologous (KO) gene families by HUMAnN v0.98. Base on the resulting KO information, MinPath was used to calculate the coverage and relative abundances of KEGG modules that are manually defined functional units [32]. Circular cladograms representing microbial taxonomic compositions and metabolic modules were implemented by using a standalone graphical tool GraPhlAn v0.9.5 (http://huttenhower.sph.harvard.edu/GraPhlAn).

### Detection of metagenomic biomarkers

In order to further test whether some taxa/metabolic modules are significantly overrepresented in the individual soil habitat, statistical analyses were performed according to the inferred relative abundances. Differentially abundant features were identified by the approach of the linear discriminant analysis (LDA) effect size (LEfSe) and could be used as metagenomic biomarkers [21]. As the sample size is not very large in this test, the significance threshold of the alpha parameter for the Krushkal-Wallis (KW) test among classes was set to 0.01 and the cut-off logarithmic LDA score was 2.0. These analyses were performed through the Galaxy server [33]. Additionally, a non-parametric test of Spearman rank correlation between the relative abundances of each KO entry and taxonomic unit was employed to estimate co-variation of community composition and functional features using the R function *cor.test*.

### Detection of microbial interactions

A recently developed computational methodology was used to investigate microbial co-occurrence and co-exclusion relationships within and between soil sites [22]. The microbial network of significant co-occurrence and co-exclusion interactions was built by a Cytoscape plugin CoNet 1.0b2 (http://psbweb05.psb.ugent.be/conet/). The taxonomic abundances estimated by MetaPhlAn were used to prepare an input matrix consisting of data from three sites (grasslands, deserts, and forest soils). The analysis was carried out with the non-default parameters listed below: 50 initial top and bottom edges; four similarity measures (Spearman, Pearson, Kullbackleibler, and Bray Curtis); edgeScores for the randomization routine; 1000 permutations and bootstraps. The resulting networks were merged based on the Simes method [34] and Benjamini-Hochberg false discovery rate (FDR) correction [35]. The FDR cutoff was set to 0.05. The ensemble co-occurrence network was visualized by Cytoscape 2.8 [36].

## Results and Discussion

### General characterization of soil community composition

**Figure 1:**
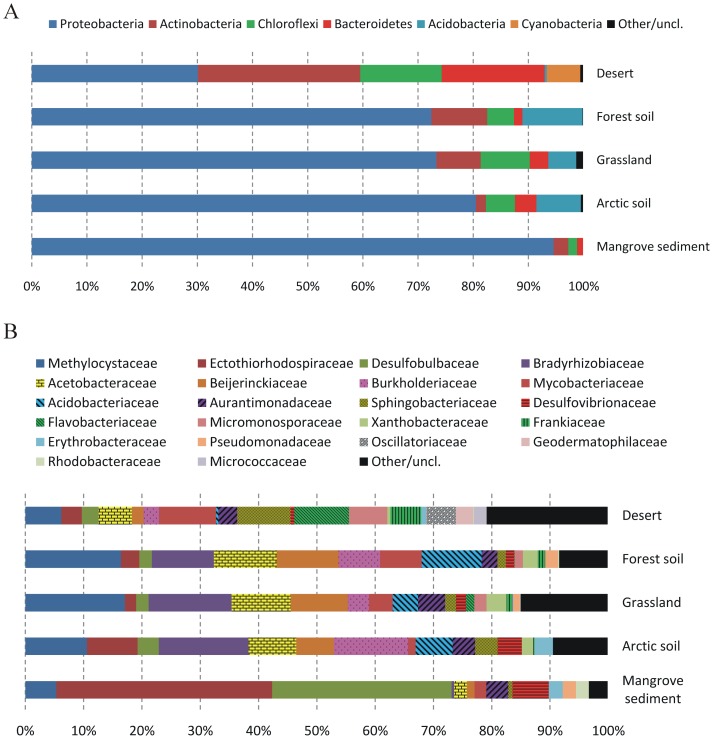
Taxonomic distribution of the soil microbial communities. A) Distribution at the phylum level; B) Distribution at the family level. Labels show the taxonomic units with average relative abundance >2% in at least one of five soil habitats: desert, forest soil, grassland, Arctic soil, and mangrove sediment.

To explore comprehensive characterizations of taxonomic compositions in the soil microbiota, 33 metagenomes sampled from five soil habitats (i.e. grassland, forest soil, desert, Arctic soil, and mangrove sediment) were included in this analysis (Table 1). Based on the assessment by MetaPhlAn, a total of 63 clades (11 phyla and 53 genera) were identified at ≥0.5% abundance in at least one sample (Table S2). Proteobacteria was the most dominated phylum in the microbial community of soil, ≥70% abundance detected in all soil sites except for the microbiota in the desert samples (Figure 1A). In desert, both phyla Proteobacteria and Actinobacteria exhibit almost identical abundance: 30% for Proteobacteria and 29% for Actinobacteria. In addition, Firmicutes and Bacteroidetes, which are the two major phyla dominating the human microbiome [37], [38], were not frequently present in the soil microbial communities. Particularly, bacterial species within the Firmicutes rarely occurred in soil. As the taxonomic distribution of environmental metagenomic sequences are greatly affected by distinct reference databases [23], the 16S amplicon approach should provide more accurate taxonomic profiling than metagenome shotgun sequencing [12]. Previous amplicon surveys of 16S rRNA gene have pointed out that bacterial phyla Acidobacteria, Actinobacteria, Bacteroidetes, Proteobacteria, and Verrucomicorbia are often abundant and ubiquitous in soil [12], [39]. Although the clade-specific marker gene database in MetaPhlAn has successfully validated the composition of the human microbiome [24], it still needs to be updated with more genomes sequenced recently from various environments.

At the family level, several families were observed to be evidently more prevalent in and specific to one soil habitat or closely related habitats (Figure 1B). For instance, the family *Methylocystaceae* was dominated and almost equally present in both sites of forest soil (16.4% abundance) and grassland (17.1%) comparing with the other three soil habitats. Additionally, among five soil habitats, both families *Ectothiorhodospiraceae* (37.0%) and *Desulfobulbaceae* (30.8%) were found to be extremely abundant in the microbiota of mangrove sediment. The enrichment of these families could be reasonably explained by the selective pressures acting on certain ecological sites. For example, organisms within *Methylocystaceae* are usually methanotrophs that can metabolize methane as their only carbon source and involved in methane oxidation [40], [41]. The DNA-level evidence identified herein may support the oxidation of methane observed in forest soil and grasslands [42], [43]. In addition, the microbiota of mangrove sediments is known to be sampled from anaerobic and hyperhaline seawater [23]. The corresponding environmental features should be beneficial for the dominance of *Ectothiorhodospiraceae* and *Desulfobulbaceae* in this particular habitat. The former comprises the most halophilic eubacteria [44] and bacteria in the latter family are strictly anaerobe sulphate reducers [45]. However, it is worth mentioning that taxonomic profiling of individual metagenome is visually distinguished from those of the other metagenomes within the same soil habitat (Figure S1). This is probably because publicly available soil metagenomes were generated by different research groups and varied in sampling strategies as well as sequencing methods. Thus, analysis of more soil metagenomes newly sequenced or coming soon is still required to statistically support the findings of soil biodiversity in the present study.

### Structure similarity and taxonomic biomarkers of soil microbial communities

**Figure 2:**
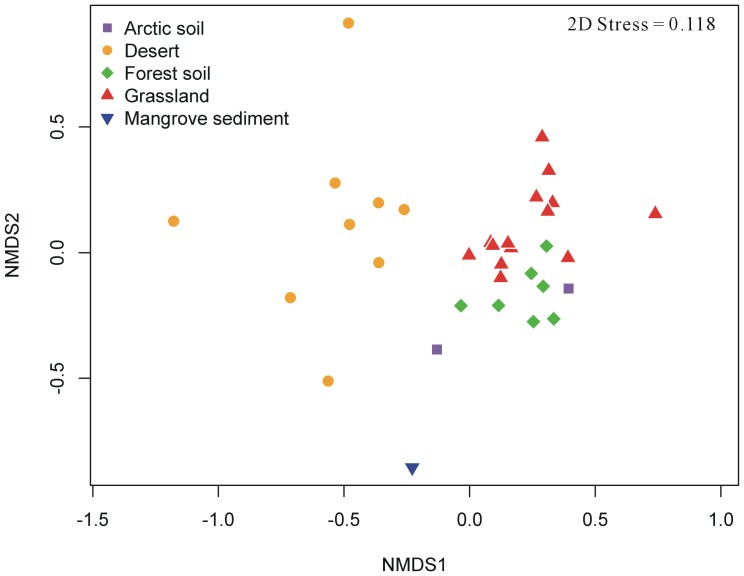
A nonmetric multidimensional scaling (NMDS) plot showing diversity of soil ecosystems. A Bray-Curtis distance similarity matrix was calculated based on the pairwise taxonomic profiles of 33 soil samples and used to generate NMDS coordinates of each sample. The distance linking two samples is shorter, indicating higher similarity between these samples. Samples from five soil sites were illustrated by different symbols and colors.

For a glimpse of structural similarity of soil microbial communities, ecological dissimilarity indices Bray-Curtis similarity scores were inferred and summarized in Table 2. The PERMANOVA test demonstrated that taxonomic compositions of microbial communities were significantly varied among soil habitats (*p* = 0.001). Meanwhile, the NMDS plot in Figure 2 further illustrated the compositional similarity among 33 samples from five soil sites. These results demonstrated that the microbiota from the same soil habitat should be more similar to each other. The community structure similarity is also influenced by varied geographical locations. E.g., the soil samples from grasslands were intensively clustered together and the corresponding similarity score (Bray-Curtis index 0.80±0.07) is indeed the highest among all inter- or intra-group comparisons (Table 2). On the contrary, the Bray-Curtis similarity score between nine desert samples is the lowest (0.58±0.16) among all intra-group comparisons, perhaps due to their sampling environments: three samples from hot deserts but the remaining ones from cold deserts [12]. Likewise, A2 sampled from the edge of the Arctic Circle is distant from A1 from high Arctic soil (Table 1). In addition, it was observed that the distances of most samples between forest soil and grasslands were closely clustered (Figure 2) and the Bray-Curtis similarity score was consistently high (0.76±0.07). Whereas, the microbiota from two extreme conditions, desert and mangrove soil, respectively, exhibited the greatest compositional dissimilarity (0.37±0.07) among all inter-group comparisons.

**Table 2:** Community structure similarity of the soil metagenomes within a habitat or between habitat pair.

Biome type	Grassland	Forest soil	Arctic soil	Desert	Mangrove soil
Grassland	**0.80±0.07**	0.76±0.07	0.68±0.09	0.50±0.12	0.45±0.02
Forest soil	0.76±0.07	**0.77**±**0.05**	0.68±0.10	0.48±0.12	0.45±0.05
Arctic soil	0.68±0.09	0.68±0.10	NA	0.45±0.11	0.52±0.15
Desert	0.50±0.12	0.48±0.12	0.45±0.11	**0.58**±**0.16**	0.37±0.07
Mangrove soil	0.45±0.02	0.45±0.05	0.52±0.15	0.37±0.07	NA

Mean and standard deviation of Bray-cutis similarities of all pairwise samples between any pair of soil habitats were shown herein. The number of sample combination less than 2 was denoted by NA.

**Figure 3:**
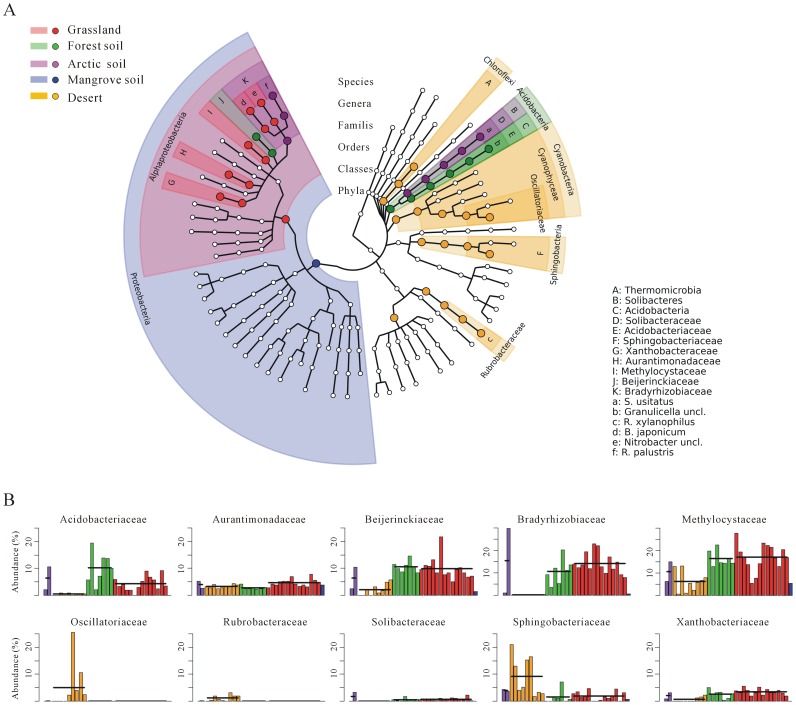
Taxonomic composition of soil microbial community based on the metagenomes from five soil habitats. A) Taxonomic cladogram showing all detected taxa (relative abundance ≥0.5%) in at least one sample. Taxonomic clades with more than five samples ≥0.5% abundance were used as inputs for LEfSe. Seven rings of the cladogram stand for domain (innermost), phylum, class, order, family, genus, and species (outermost), respectively. Enlarged circles in color are the differentially abundant taxa identified to be metagenomic biomarkers and the circle color is corresponding to the individual soil habitat in which the taxon is the most abundant among 5 soil ecosystems (Green for forest soil, red for grassland, purple for Arctic soil, blue for mangrove sediment, and orange for desert). B) The histograms of relative abundances of family-level biomarkers in each sample. Bacterial families significantly differential among all pairwise comparisons were illustrated. The average abundance of each family in the individual soil habitat was denoted by the horizontal line.

To further investigate the taxonomic distribution and differentially abundant clades of diverse soil ecosystems, we compared the abundances of microbial compositions at each taxonomic level. Figure 3A shows a cladogram visualizing all detected microbial compositions (≥0.5% abundance) from domain to species, respectively. Based on the inferred taxonomic profiling of all samples, a statistical strategy for discovering metagenomic biomarkers was carried out by LEfSe and determined 46 differentially abundant taxa (Table S2). Among these differentially abundant taxa, 10 and 12 were found to be family- and genus-level biomarkers, respectively (Figure 3A). These detected taxonomic biomarkers could be used as candidate indicators to distinguish at least one microbial community of five individual soil habitats. E.g., two families *Beijerinckiaceae* and *Methylocystaceae* that consist of methanotrophic taxa [46] were detected to be family-level biomarkers (*P* value <0.01) that were most abundant in the forest soil and grassland, respectively. The abundances of both families were found to be significantly decreased in desert and mangrove sediment (Figure 3B). The abundance differences of these methanotrophs might be positively associated with the expected capability of methane oxidation among distinct soil ecosystems. Although the organisms within the *Alphaproteobacteria* class were most differentially abundant in the grassland community, a genus-level biomarker within *Alphaproteobacteria* was specially enriched in the communities of forest soil and Arctic soil, respectively (Figure 3A). Intriguingly, the desert community had two phylum-level markers, Cyanobacteria and Chloroflexi, both of which showed the highest abundance in deserts comparing with other soil sites (Figure 3A). Bacteria in both phyla can produce their energy through photosynthesis [47]. It was worth noting that the family Oscillatoriaceae within Cyanobacteria was significantly enriched in the desert microbial community (Figure 3B). The enrichment of these bacterial groups should be consistent with the following environmental features of deserts: extreme arid, strong light, and poor nutrient conditions. In addition, six species were found to be differentially abundant, some of which were uniquely present in the individual soil habitat. E.g., *Rubrobacter xylanophilus* only occurs in the microbiota of deserts. *R. xylanophilus* is the most thermophilic actinobacterium known and bears extreme tolerance to desiccation [48]. *Bradyrhizobium japonicum*, an agriculturally important species of legume-root nodulating [49], was found to be the most abundant in grassland and second in forest soil.

### Metabolic potential and functional biomarkers of soil microbial communities

**Figure 4:**
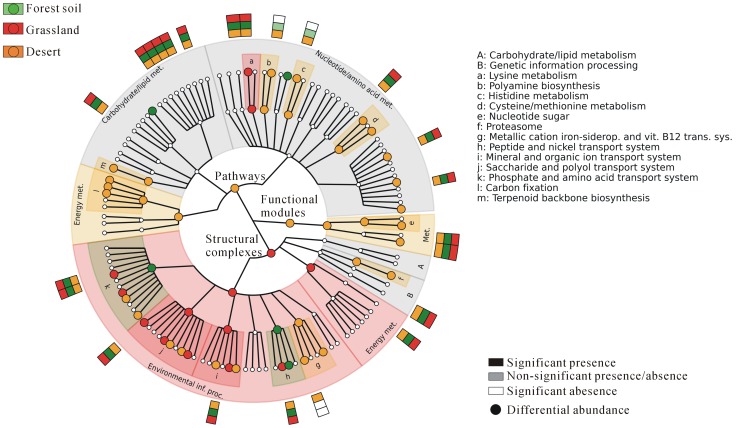
Metagenome-level metabolic reconstruction of the soil microbial community. KEGG BRITE hierarchical structures that are illustrated by the innermost four rings were used to cluster metabolic modules. The outermost ring composed of circles denotes KEGG functional modules detected in at least one of 30 metagenomes from three soil sites. Differentially abundant modules were inferred by LEfSe and illustrated by the enlarged circles in distinct colors: green stands for the modules most abundant in the forest soil, red for the grassland, and orange for the desert. The outermost rectangles denote core and differentially covered modules among three soil sites: ≥90% coverage stands for presence and ≤10% coverage for absence.

Besides microbial composition, metabolic potential of soil microbial communities was also investigated. In this study, we focused on the KEGG modules that are tight functional units composed of approximately 5 to 20 genes and beneficial for biological interpretation of metagenomes [29], [30], [38]. To further enhance the performance of statistical inference on the functional analysis, two soil sites with limited samples (two samples from Arctic soil and one sample from mangrove sediment) were excluded. After translated BLAST searching against the database of KO gene families, we found an average of ∼33.6% of reads mapped to at least one KO entry (Table S3). Based on the metabolic reconstruction of 30 metagenomic datasets using HUMAnN, Figure 4 shows 119 functional modules detected in the microbial communities of grasslands, forest soils, and deserts (Table S4). Of these functional modules, we found 20 core metabolic modules that were almost entirely present at >90% coverage in all soil metagenomes tested (Table 3). Some of these core modules were essential for basic life activities of prokaryotic cells in soil, such as central carbon metabolism (M00002-3, M00007, M00009, M00011-12), nucleotide and amino acid metabolism (M00016, M00018, M00048, M00115, M00125), translation (M00178, M00359-360), and ATP synthesis (M00144). In addition, all the remaining core modules were involved in certain transport systems, three (M00207, M00222, M00239) of which are also detected in the core modules of the human microbiome [29]. On the other hand, three functional modules (M00026, M00133, M00319) were differentially covered among three soil sites (Figure 4; Table S5). It was worth noting that structural complex module manganese/zinc/iron transport system (M00319) was completely present only in the deserts but appeared to be absent in both grasslands and forest soils. It indicates that deserts microbiota is well-equipped with metal acquisition systems that play potential roles in the maintenance of metal homeostasis [50].

**Table 3:** Core metabolic modules shared by grasslands, deserts, and forest soils.

Module ID	Definition of modules in KEGG
M00002	Glycolysis, core module involving three-carbon compounds
M00003	Gluconeogenesis, oxaloacetate = > fructose-6P
M00007	Pentose phosphate pathway, non-oxidative phase, fructose 6P = > ribose 5P
M00009	Citrate cycle (TCA cycle, Krebs cycle)
M00011	Citrate cycle, second carbon oxidation
M00012	Glyoxylate cycle
M00016	Lysine biosynthesis, aspartate = > lysine
M00018	Threonine biosynthesis, apartate = > homoserine = > threonine
M00048	Inosine monophosphate biosynthesis, PRPP + glutamine = > IMP
M00115	NAD biosynthesis, aspartate = > NAD
M00125	Riboflavin biosynthesis, GTP = > riboflavin/FMN/FAD
M00144	Complex I (NADH dehydrogenase), NADH dehydrogenase I
M00178	Ribosome, bacteria
M00185	Sulfate transport system
M00207	Multiple sugar transport system
M00222	Phosphate transport system
M00237	Branched-chain amino acid transport system
M00239	Peptides/nickel transport system
M00359	Aminoacyl-tRNA biosynthesis, eukaryotes
M00360	Aminoacyl-tRNA biosynthesis, prokaryotes

Furthermore, 33 functional modules were detected to be differentially abundant in at least one of three soil sites (Figure 4 and Table S4). Interestingly, two thirds of these modules were significantly enriched in the microbiota of deserts in comparison to the microbiota of grasslands and forest soils. Of them, three metabolic modules (M00165-167) are involved in the reductive pentose phosphate cycle (Benson-Calvin cycle), which is the main pathway for the conversion of atmospheric CO_2_ to organic compounds [51]. It was worth noting that these overrepresented modules involved in carbon fixation might be consistent with high abundance of photosynthetic organisms Cyanobacteria present in the microbiota of desert. Additionally, eight structural complex modules detected to be functional biomarkers in deserts are responsible for the transport of metallic cation (M00317, M00319), mineral and organic ion (M00321, M00299), saccharide and polyol (M00201, M00199), glutamate (M00233), and urea (M00323). On the other hand, we found that two metabolic modules (M00022: Shikimate pathway and M00237: Branched chain amino acid transport system) were significantly overrepresented in grasslands and forest soils comparing with plant-free deserts (Figure 4 and Table S4). Both modules are associated with plant-derived metabolites [52], [53]. These results showed that some modular metabolic activities are likely to be associated with the individual soil ecosystem. However, more metagenome samples from different sites are needed for accurately statistical validation of these characterized modules as promising biomarkers for diverse soil communities.

### Correlation between microbial compositions and functions

Similar to the approach presented by Segata et al. [38], we assessed the correlations between microbial compositional and functional enrichment. The results showed that some significant associations between taxonomic clades and functional gene families were detected in the soil microbial communities (Spearman non-parametric test; Benjamini-Hochberg corrected *p*-value <0.01) (Figure S2). Notably, several taxonomic biomarkers possessed by individual microbial community mentioned above were further confirmed by the related strong associations between gene families and taxonomic clades. E.g. the gene *petA* (K02634) encoding apocytochrome f protein involved in photosynthesis was positively associated with the members of Cyanobacteria (Spearman test; *q*-value <0.001), one of the earliest prokaryotic organisms which can carry out oxygenic photosynthesis on Earth [47]. In addition, a significantly positive correlation (Spearman test; *q*-value <0.001) between methanotrophs *Methylocystaceae* and the gene *mcl* (K08691) coding for malyl-CoA lyase was observed in the microbial community of grassland. The enrichment of protein Mcl involved in both pathways of methane metabolism and carbon fixation, should be consistent with the featured metabolic activities of these methanotrophs.

### Soil microbial interaction network

**Figure 5:**
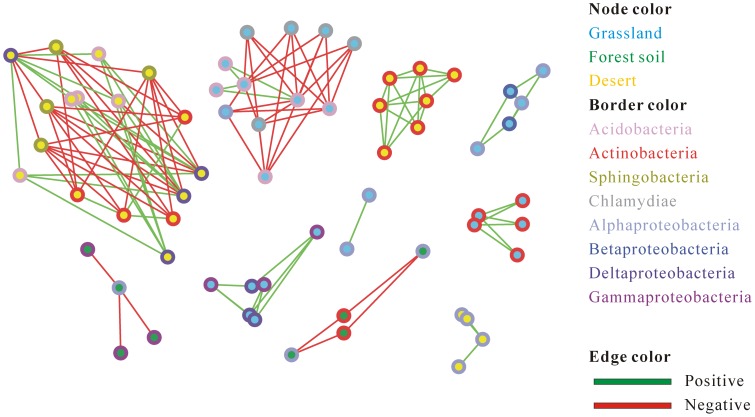
A global microbial interaction network of the soil microbial community. The network captured all significant associations (multiple corrected *p*-value <0.05) among the abundances of phylotypes at or above the genus level in the soil microbial community within and across the three soil sites. Phylotypes were illustrated by nodes (light blue for grasslands, blue for forest soils, and yellow for deserts) and edges denote significant correlations between phylotypes: positive correlation colored in green means co-occurrence whereas negative correlation in red means mutual exclusion. The border of nodes was colored according to taxonomic affiliations at the class level.

To further decipher complex ecological relationships in the individual soil microbial community, microbial association networks were inferred based on the estimated taxonomic profiling. In this case, we intended to focus on the microbial associations within the single soil habitat, i.e. forest soil, grassland, and desert. The resultant metagenome-wide networks comprised 126 significant associations among 66 phylotypes at or above the genus level (Benjamini-Hochberg corrected *p*-value <0.05) (Figure 5). Of these significant phylotype correlations, 54% was detected to be co-present and the remaining was mutually excluded. Interestingly, we found that three quarters (∼74%) of co-occurrence patterns were constituted by the taxa within the same phyla; whereas nearly all co-exclusion patterns (∼90%) consisted of the taxa from the distinct phyla. The evidence presented herein can again support the previous notion that phylotypes with closely evolutionary relationships usually tend to co-occur [8]. E.g., three families (*Bifidobacteriaceae*, *Mycobacteriaceae*, and *Frankiaceae*) belonging to the same class Actinobacteria showed pairwise positive correlation in the microbiota of desert (Figure 5). Similar taxon co-occurrence pattern was also found between *Bifidobacteriaceae* and *Frankiaceae* in the microbiota of grassland. Additionally, two genera within the family *Bradyrhizobiaceae* co-occurred in the grassland community: one is nitrogen-fixing bacteria *Nitrobacter* and the other is phototrophic bacteria *Rhodopseudomonas*. On the other hand, those mutually excluded bacteria were found to be evolutionarily unrelated. E.g., *Sphingobacteriaceae* belonging to the Bacteroidetes were negatively associated with *Desulfovibrionaceae* from the Proteobacteria and *Rubrobacteraceae* from the Actinobacteria in the microbiota of desert (Figure 5). Although most phylotype associations in the network lack empirical evidence to support their natural presence, it provides some promising targets at least to shed light on the complex cooperative or competitive mechanisms among soil microorganisms.

## Conclusions

In this study, comparative metagenomic characterizations of divergent soil microbial communities were described in details by an integrated bioinformatics analysis pipeline. Complicated phylogenetic and metabolic networks with a spectrum of taxonomic and functional biomarkers were comprehensively illustrated at the metagenome level for soil. Cooperative or competitive associations among microbes from diverse soil ecosystems were also inferred to understand complex microbial interactions in the soil metagenome. This study provides new insights into the relation between soil biodiversity and ecosystem function, and provides applicable analysis and visualization approaches for studying soil microbial communities.

## Supporting Information

Figure S1 **Taxonomic distribution of 33 metagenomes from soil microbial communities.** A) Distribution at the phylum level; B) Distribution at the family level. Labels show the taxonomic units with average relative abundance >2% in at least one of 33 samples.
(TIF)

Figure S2 **Co-variation of bacterial clades and KEGG orthologous gene families in the desert microbiome.** The spearman non-parametric correlation of each KEGG gene family against each taxonomic clade was assessed. After multiple testing corrections based on the Benjamini-Hochberg procedure, a network of significant correlations between gene families and taxonomic clades was shown herein (q-value <0.01). Ellipses denote taxa and rectangles stand for KEGG gene families. The edge linking taxonomic clade and gene family indicates that strong correlation was detected in the individual microbial community: green for forest soil, red for grassland, and orange for desert.
(TIF)

Table S1 Sequence data accession numbers and/or web links of soil metagenomes used in this study.
(XLSX)

Table S2 Taxonomic profiling of the soil metagenomes estimated in this study. Relative abundances of taxa were inferred by MetaPhlAn. Differentially abundant clades among five soil habitats were detected by LEfSe and labeled by the soil site with the highest LDA score among pairwise comparisons of all sites. According to the non-strict and strict statistical strategy, 46 taxonomic biomarkers were detected to be significantly differential in at least one of five soil habitats.
(XLSX)

Table S3 The proportion of reads mapped to MetaPhlAn clade-specific marker genes and KEGG orthologous gene families.
(XLSX)

Table S4 Estimated values for relative abundances of KEGG functional modules in the soil microbial community. Differentially abundant modules were detected by LEfSe and labeled by the soil habitat with the highest LDA score among pairwise comparisons of all habitats.
(XLSX)

Table S5 Estimated values represented by percentage of the coverage of KEGG functional modules in the soil microbial community. The presence/absence of modules was defined as follows: the median of coverage estimates of the samples per site >0.9 stands for presence; the median <0.1 for absence.
(XLSX)
