# Supplementary figures and images for: Bioinformatic Approaches Reveal Metagenomic Characterization of Soil Microbial Community
Source: PLoS One. 2014 Apr 1;9(4):e93445. doi: 10.1371/journal.pone.0093445 (PMC3972102; doi:10.1371/journal.pone.0093445)

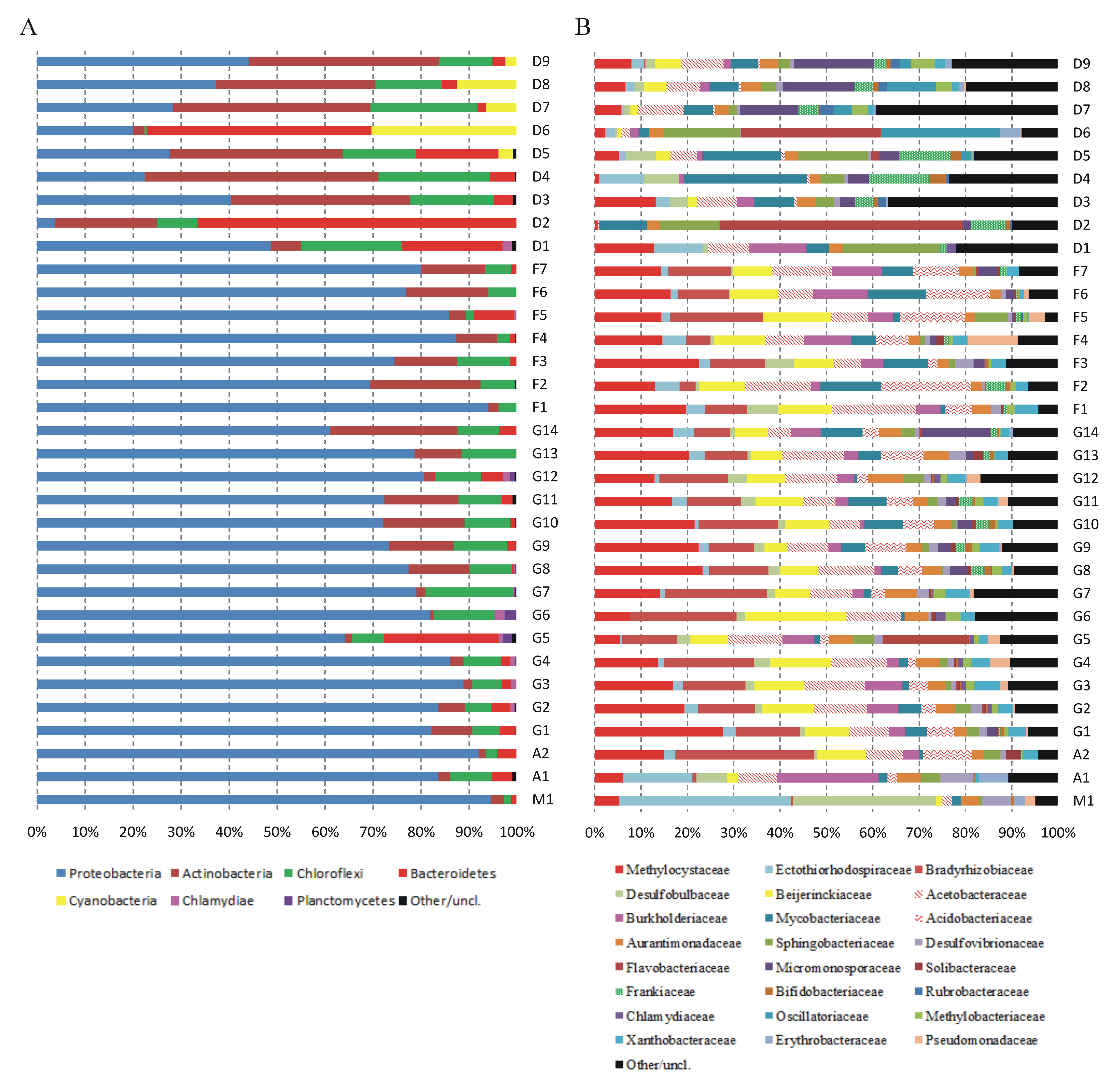

Supplement: Figure S1 — Taxonomic distribution of 33 metagenomes from soil microbial communities. A) Distribution at the phylum level; B) Distribution at the family level. Labels show the taxonomic units with average relative abundance >2% in at least one of 33 samples. (TIF) [file pone.0093445.s001.tif]

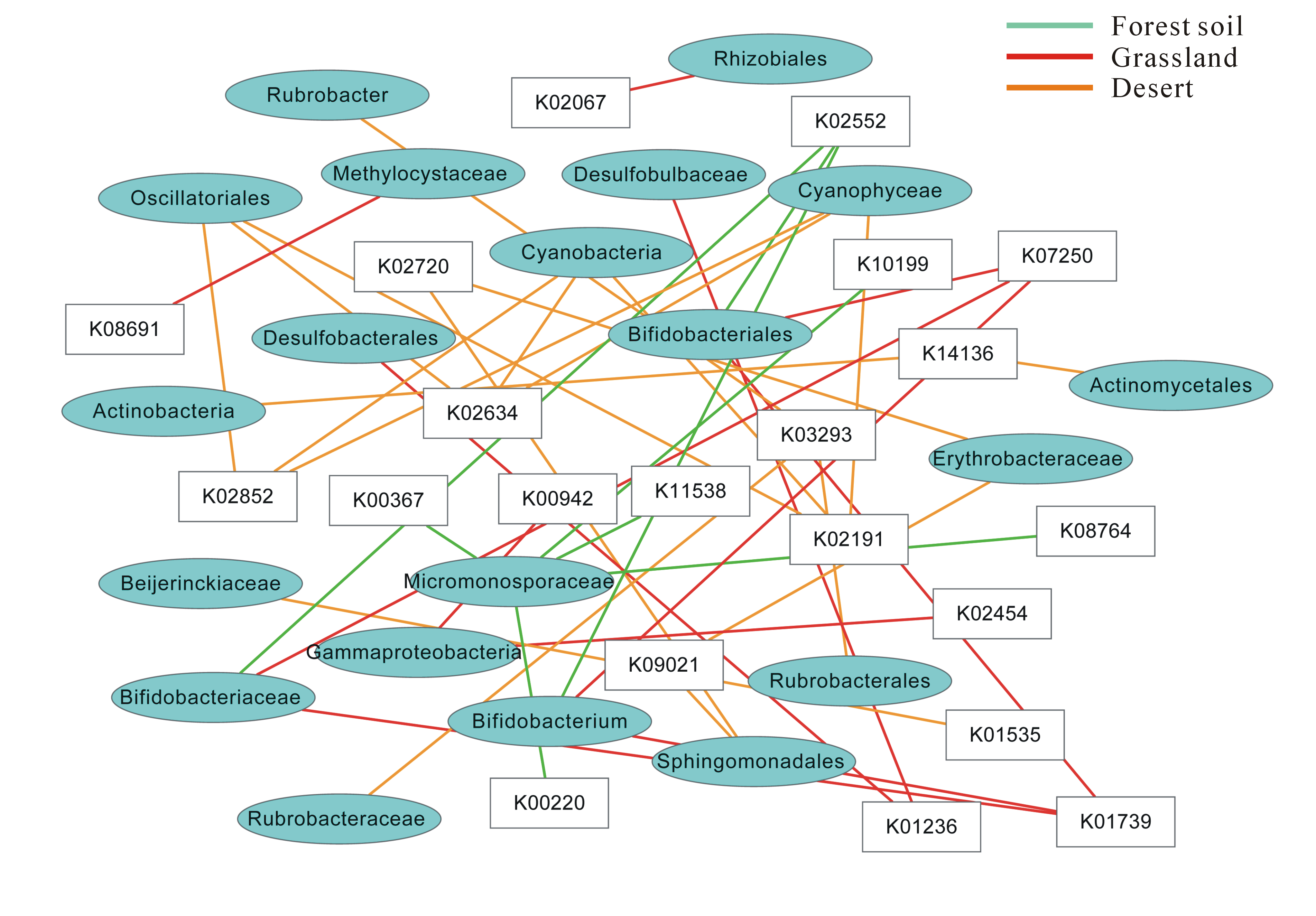

Supplement: Figure S2 — Co-variation of bacterial clades and KEGG orthologous gene families in the desert microbiome. The spearman non-parametric correlation of each KEGG gene family against each taxonomic clade was assessed. After multiple testing corrections based on the Benjamini-Hochberg procedure, a network of significant correlations between gene families and taxonomic clades was shown herein (q-value <0.01). Ellipses denote taxa and rectangles stand for KEGG gene families. The edge linking taxonomic clade and gene family indicates that strong correlation was detected in the individual microbial community: green for forest soil, red for grassland, and orange for desert. (TIF) [file pone.0093445.s002.tif]
